# *In vivo* imaging of T cell lymphoma infiltration process at the colon

**DOI:** 10.1038/s41598-018-22399-2

**Published:** 2018-03-05

**Authors:** Yoshibumi Ueda, Toshiyuki Ishiwata, Seiichi Shinji, Tomio Arai, Yoko Matsuda, Junko Aida, Naotoshi Sugimoto, Toshiro Okazaki, Junichi Kikuta, Masaru Ishii, Moritoshi Sato

**Affiliations:** 1 Graduate School of Arts and Sciences, The University of Tokyo, 3-8-1 Komaba, Meguro-ku, Tokyo 153-8902 Japan; 2 AMED-PRIME, Japan Agency for Medical Research and Development, Tokyo, Japan; 3 Division of Aging and Carcinogenesis, Research Team for Geriatric Pathology, Tokyo Metropolitan Institute of Gerontology, Tokyo, 173-0015 Japan; 4 Department of Gastrointestinal and Hepato-Biliary-Pancreatic Surgery, Nippon Medical School, Tokyo, 113-8603 Japan; 5 Department of Pathology, Tokyo Metropolitan Geriatric Hospital, Tokyo, 173-0015 Japan; 6 Department of Physiology, Graduate School of Medical Science, Kanazawa University, Kanazawa, Ishikawa, Japan; 7 Department of Hematology and Immunology, Kanazawa Medical University, 1-1 Daigaku, Uchinada, Ishikawa, 920-0293 Japan; 8 Department of Immunology and Cell Biology, Graduate School of Medicine and Frontier Biosciences, Osaka University, 2-2, Yamada-oka, Suita, Osaka Japan

## Abstract

The infiltration and proliferation of cancer cells in the secondary organs are of great interest, since they contribute to cancer metastasis. However, cancer cell dynamics in the secondary organs have not been elucidated at single-cell resolution. In the present study, we established an *in vivo* model using two-photon microscopy to observe how infiltrating cancer cells form assemblages from single T-cell lymphomas, EL4 cells, in the secondary organs. Using this model, after inoculation of EL4 cells in mice, we discovered that single EL4 cells infiltrated into the colon. In the early stage, sporadic elongated EL4 cells became lodged in small blood vessels. Real-time imaging revealed that, whereas more than 70% of EL4 cells did not move during a 1-hour observation, other EL4 cells irregularly moved even in small vessels and dynamically changed shape upon interacting with other cells. In the late stages, EL4 cells formed small nodules composed of several EL4 cells in blood vessels as well as crypts, suggesting the existence of diverse mechanisms of nodule formation. The present *in vivo* imaging system is instrumental to dissect cancer cell dynamics during metastasis in other organs at the single-cell level.

## Introduction

The infiltration and growth of cancer cells in secondary organs are of great interest because they play an important role in the formation of potentially fatal metastatic foci. Until formation of metastatic foci, cancer cells undergo a series of sequential steps, including survival in the circulation against functional host immunity, infiltration of single cancer cells into target organs via the blood or lymph vessels, extravasation, and finally, initiation of proliferation^1^. Although understanding each stage of metastasis is important from the perspective of drug development and therapeutics, knowledge remains limited.

Fluorescence microscopy is often employed to observe metastasis^2–4^. Several reports described detection of liver, lung, and brain metastasis after fluorescent cancer cells were grafted onto the ovary or injected into the tail vein in mice^2,5,6^. However, low fluorescence intensity of cancer cells can make it difficult to visualize single cells with subcellular resolution^2,5,7,8^. This issue hampers efforts to identify the sites at which single cancer cells infiltrate in the initial step of metastasis.

In order to overcome these issues, we prepared EL4 cells, which are mouse malignant T-cell lymphoma cells that stably express EGFP and DsRed2. The fluorescence emitted by EGFP- and DsRed2-positive cells is three and greater than two orders of magnitude more intense, respectively, than auto-fluorescence. Therefore, we were able to observe cancer cell dynamics at subcellular resolution, even *in vivo*. Using these cells in combination with two-photon microscopy, we observed the infiltration of single EL4 cells in the epithelial layer of the mucosa of the colon. We also describe the dynamics of single EL4 cells with subcellular resolution and the diversity of EL4 cell assemblage formations during the initial steps of metastatic foci establishment.

## Results

### Lodging of single EL4-EGFP cells in the blood vessels adjacent to crypts in the colon

**Figure 1:**
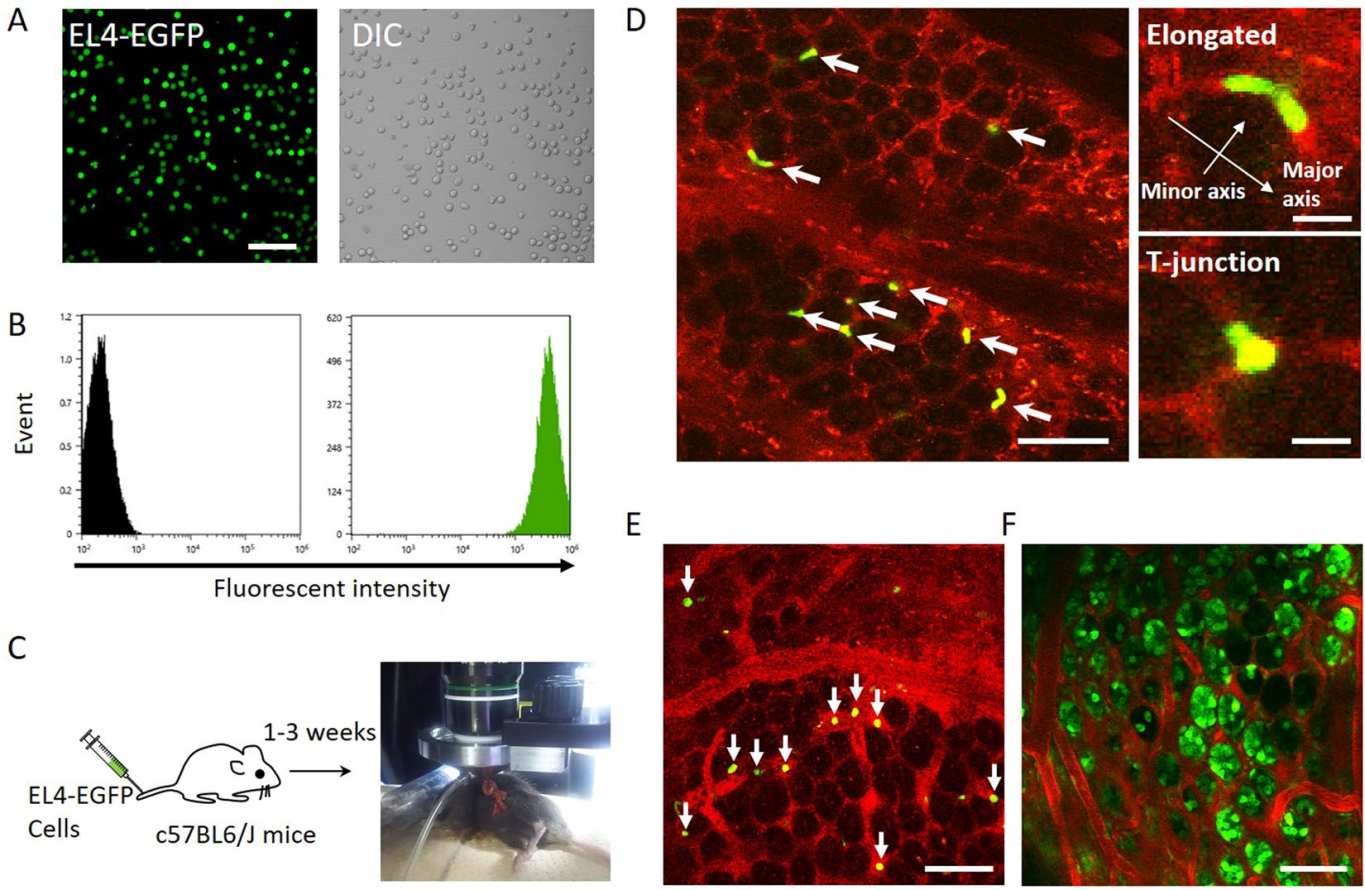
Localization of EL4-EGFP cells in the blood vessels adjacent to crypts in the colon of C57BL6/J mice. (**A**) EL4 cells stably expressing EGFP *in vitro* under fluorescence microscopy (LSM710, Carl Zeiss). Bar indicates 50 μm. (**B**) EGFP fluorescence intensity measured using a cell analyzer (SH800, Sony). (**C**) At 1 to 3 weeks after EL4-EGFP cell injection, the colon was removed from the body and observed on living tissues. (**D**) EL4-EGFP cell imaging of in the colon. Green color indicates EGFP of EL4-EGFP cells, as shown with white arrows. Blood vessels were stained with rhodamine B–conjugated dextran (M.W. 70,000) (red). Bar indicates 50 μm. Right upper panel shows an enlarged image of an elongated EL4 cell. Bar indicates 10 μm. Right lower panel shows an EL4 cell lodged in the T-junction of blood vessels. Bar indicates 10 μm. These images were representative from 3 mice examined. (**E**). Imaging of EL4-EGFP cells localized in large blood vessels under the crypts in the mucosal layer. EL4-EGFP cells are indicated by white arrows. Bar indicates 50 μm. (**F**). Crypts visualized using green mice (C57BL/6-Tg[CAG-EGFP]). Crypts and blood vessels are shown in green and red, respectively. Bar indicates 50 μm.

First, we characterized EL4 cells stably expressing EGFP (Fig. 1A). More than 99% of the EL4 cells were EGFP-positive (Fig. 1B). The EGFP fluorescence intensity of the EL4 cells was 1,000 times greater than the auto-fluorescence (Fig. 1B). The fluorescence intensity remained at the same level during the course of this study. The fluorescence properties of the EL4 cells were therefore suitable for *in vivo* imaging, and the cells were designated EL4-EGFP.

Next, EL4-EGFP cells were injected into the tail vein of C57BL6/J mice. At day 7 to 14 after injection (the early stage), the mice were anesthetized, rhodamine B isothiocyanate–dextran was injected into the tail vein to visualize the blood vessels, the abdomen was opened, and the colon was removed from the body (Fig. 1C) and observed under a two-photon microscope. We found EL4 cells lodged in small blood vessels such as the capillaries (diameter 3–8 μm) in the mucosal layer (Fig. 1D, white arrows) and cells flowing in the large blood vessels under the mucosal layer (Fig. 1E, white arrows). To identify the colon structures adjacent to the EL4 cells, EGFP-expressing mice (C57BL/6-Tg[CAG-EGFP]; ‘green mice’) were used. Characteristic crypt structures were highlighted by EGFP adjacent to the blood vessels (Fig. 1F), indicating that EL4 cells were localized in the blood vessels adjacent to the crypts.

### Lodging of single EL4-DsRed2 cells in blood vessels adjacent to crypts in the colon of EGFP-expressing mice

**Figure 2:**
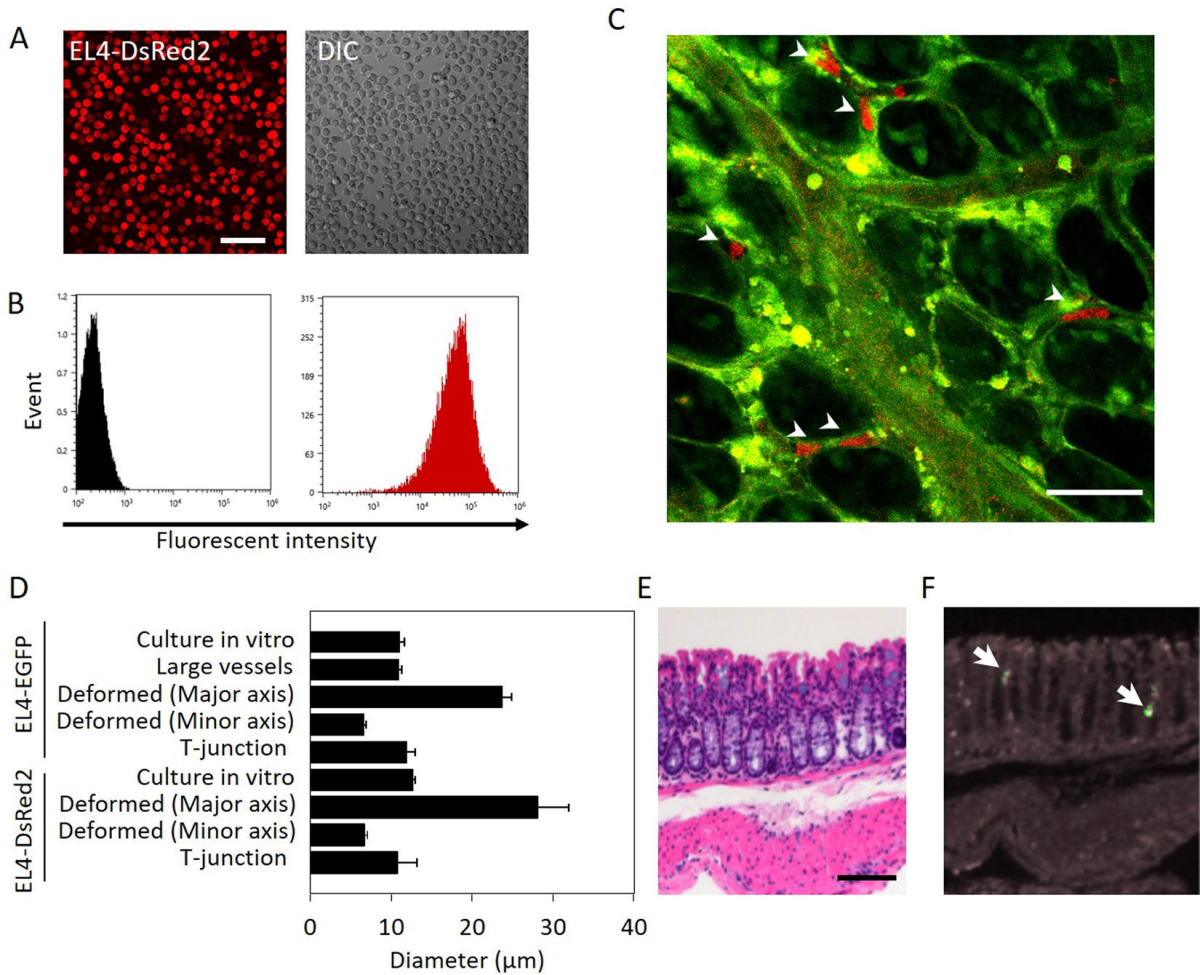
Localization of EL4-DsRed2 cells in blood vessels adjacent to crypts in the colon of green mice. (**A**) EL4 cells stably expressing DsRed2 *in vitro* observed under a fluorescence microscope. Bar indicates 50 μm. (pCAG-DsRed2 sequence is in supplementary data2). (**B**) DsRed2 fluorescence intensity measured using a cell analyzer (SH800, Sony). (**C**) Imaging of EL4-DsRed2 cells in the colon. Red color indicates DsRed2 in EL4-DsRed2 cells, as shown by white arrowheads. Green color mainly shows endothelial cells of blood vessels. Bar indicates 50 μm. This image was representative from 4 mice examined. (**D**) Analyses of the diameter of EL4-EGFP cells *in vitro* and in colonic blood vessels. Error values and bars indicate standard error (S.E.). (**E**) Hematoxylin and eosin staining and fluorescence analysis of the infiltration of EL4 cells into colon tissues. EL4 cells (5 × 10^5^) were injected into 9-week-old C57BL6/J mice via the tail vein. The colon was removed and fixed with 4% paraformaldehyde at 14 days after EL4 cell injection. The colon tissue was stained with hematoxylin and eosin. Bar indicates 50 μm. (**F**) Infiltration of EL4 cells was also examined by fluorescence microscopy with the same sample used in (**E**).

In order to examine EL4 cell localization with different color-positive EL4 cells, a line of DsRed2-expressing EL4 cells was established (EL4-DsRed2 cells) (Fig. 2A). More than 98% of these EL4 cells were DsRed2-positive (Fig. 2A). The fluorescence intensity of DsRed2 in the EL4 cells was 400 times greater than the autofluorescence (Fig. 2B) and remained stable during the course of this study. EL4-DsRed2 cells were injected into green mice via the tail vein. In the early stage, EL4-DsRed2 cells were also observed in the blood vessels adjacent to the crypts, as was the case with EL4-EGFP cells (Fig. 2C). After analyses of the localization and morphology of EL4-EGFP and EL4-DsRed2 cells, the cells were classified into three types. The first type included cells elongated along the small blood vessels (Fig. 1D, right upper panel, and 2C). The lengths of the major and minor axes of the deformed EL4-EGFP cells were 23.8 ± 1.12 and 6.85 ± 0.27 μm, respectively, indicating that the EL4-EGFP cells were 2.16 times longer in the direction of the major axis compared with EL4 cells examined *in vitro* (11.03 ± 0.29 μm) (Fig. 2D). In the case of EL4-DsRed2 cells, the lengths of the major and minor axes were 28.13 ± 3.77 and 6.63 ± 0.47 μm, respectively, indicating that the EL4-DsRed2 cells were 2.21 times longer in the direction of the major axis compared with EL4-DsRed2 cells examined *in vitro* (12.7 ± 0.20 μm) (Fig. 2D). The second type included EL4 cells localized at T-junctions of small blood vessels (Fig. 1D, right lower panel). The diameter of these cells (EL4-EGFP: 12.0 ± 0.99 μm, EL4-DsRed2: 10.8 ± 2.37 μm) was similar to that of cells examined *in vitro*. The third type included EL4 cells flowing in the large blood vessels under the mucosal layer (Fig. 1E) (11.57 ± 0.17 μm), and the diameter of these cells was also similar to that of cells examined *in vitro*. Collectively, these results show that EL4 cells infiltrate into the blood vessels in the mucosal layer of the colon and elongate in the small blood vessels, behaviors that were not observed *in vitro*.

Next, EL4 cell infiltration was investigated histologically using hematoxylin and eosin (HE) staining of 4% paraformaldehyde–fixed paraffin-embedded colon tissue samples. The colon tissues were prepared from mice 14 days after inoculation with EL4-EGFP cells. Infiltration of single EL4 cells could not be detected histologically in these tissue samples by two pathologists (T.I. and Y.M., Fig. 2E). When we attempted to detect fluorescence from these samples using fluorescence microscopy, only a few cells were observed (Fig. 2F, white arrows). In addition, it was not possible to discern the details of the shape of the EL4 cells at subcellular resolution.

### EL4 cell dynamics in colonic blood vessels

**Figure 3:**
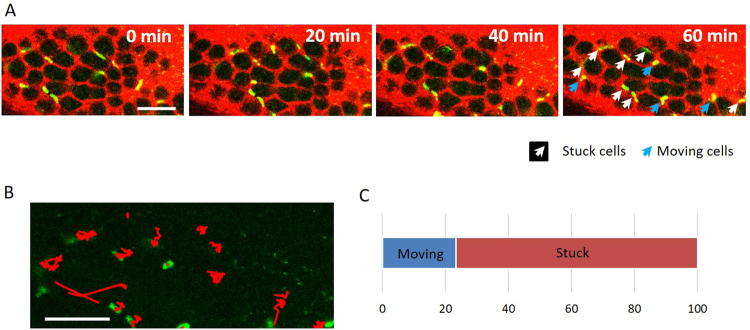
Time-lapse imaging of EL4-EGFP cells in colonic blood vessels. (**A**) Time-lapse (20-min intervals) imaging of EL4-EGFP cells (green) in small blood vessels (red). EL4-EGFP cells (5 × 10^5^) were injected into the tail vein of C57BL6/J mice and observed 10 days after injection. White arrows show lodged EL4-EGFP cells. Blue arrows indicate moving EL4 cells. Bar indicates 100 μm. (**B**) Tracking analysis of EL4-EGFP cell movement for 1 h. Traces of moving cells are shown in red. Bar indicates 100 μm. (**C**). Ratio of moving and lodged cells determined at 20-min intervals. A total of 23.3 ± 0.32% of the EL4 cells exhibited motility. Analyses were performed from 3 mice studied in the early stage of metastasis.

**Figure 4:**
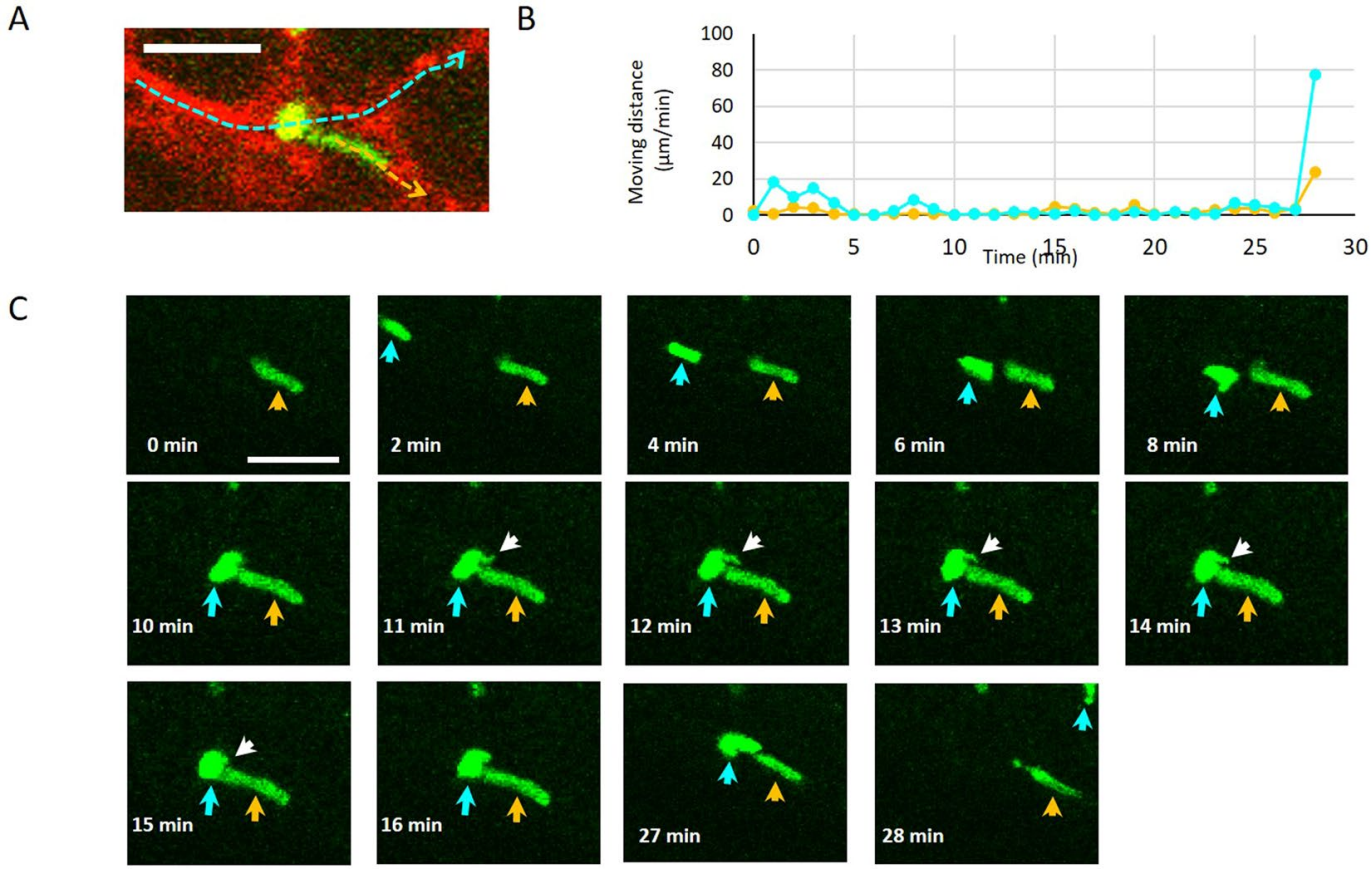
Time-lapse imaging of highly motile EL4-EGFP cells in colonic blood vessels. (**A**) Dynamics of EL4 cells examined at subcellular resolution. Migration of two EL4 cells is shown with blue and orange lines. Bar indicates 50 μm. (**B**) Analysis of distance traversed by EL4 cells each min. (**C**). Observation of two individual EL4 cells in small vessel in the colon. Note that we observed a pseudopod (white arrow) extending from one EL4 cell (blue arrow) and touching the other cell (orange arrow). Bar indicates 50 μm.

Next, real-time imaging was carried out to investigate the dynamics of EL4 cells in the blood vessels of the colon. Although more than 70% of the EL4 cells were lodged in the blood vessels even after 1 h of observation (Fig. 3A [white arrows], 3B, and Supplementary Movie S1), other EL4 cells moved within the blood vessels (Fig. 3A [blue arrows], 3B, and Supplementary Movie S1). Analyses at 20-min intervals showed that 23.3 ± 0.32% of the EL4 cells were motile (Fig. 3C). We then focused on the moving cells and examined the characteristics of their motility. Two moving cells were observed for 28 min (Fig. 4A). Analysis of the distance moved over 1-min intervals revealed that the cells moved irregularly (Fig. 4B). In the first 8 min, one EL4 cell (blue arrow) moved through a small blood vessel and clogged it with another cell (orange arrow) that was lodged in the blood vessel (Fig. 4C and Supplementary Movie S2). While lodged in the clogged blood vessel, the cell denoted with a blue arrow extended a pseudopod (white arrow) and touched the other cell. At 27 min after clogging the blood vessel, the cells simultaneously moved away. These data indicate that EL4 cells are highly motile and can interact with other cells. Additionally, our system allows us to observe the detailed morphologic changes of cells *in vivo*.

### Formation of small nodules composed of several EL4-EGFP cells in crypts and blood vessels

**Figure 5:**
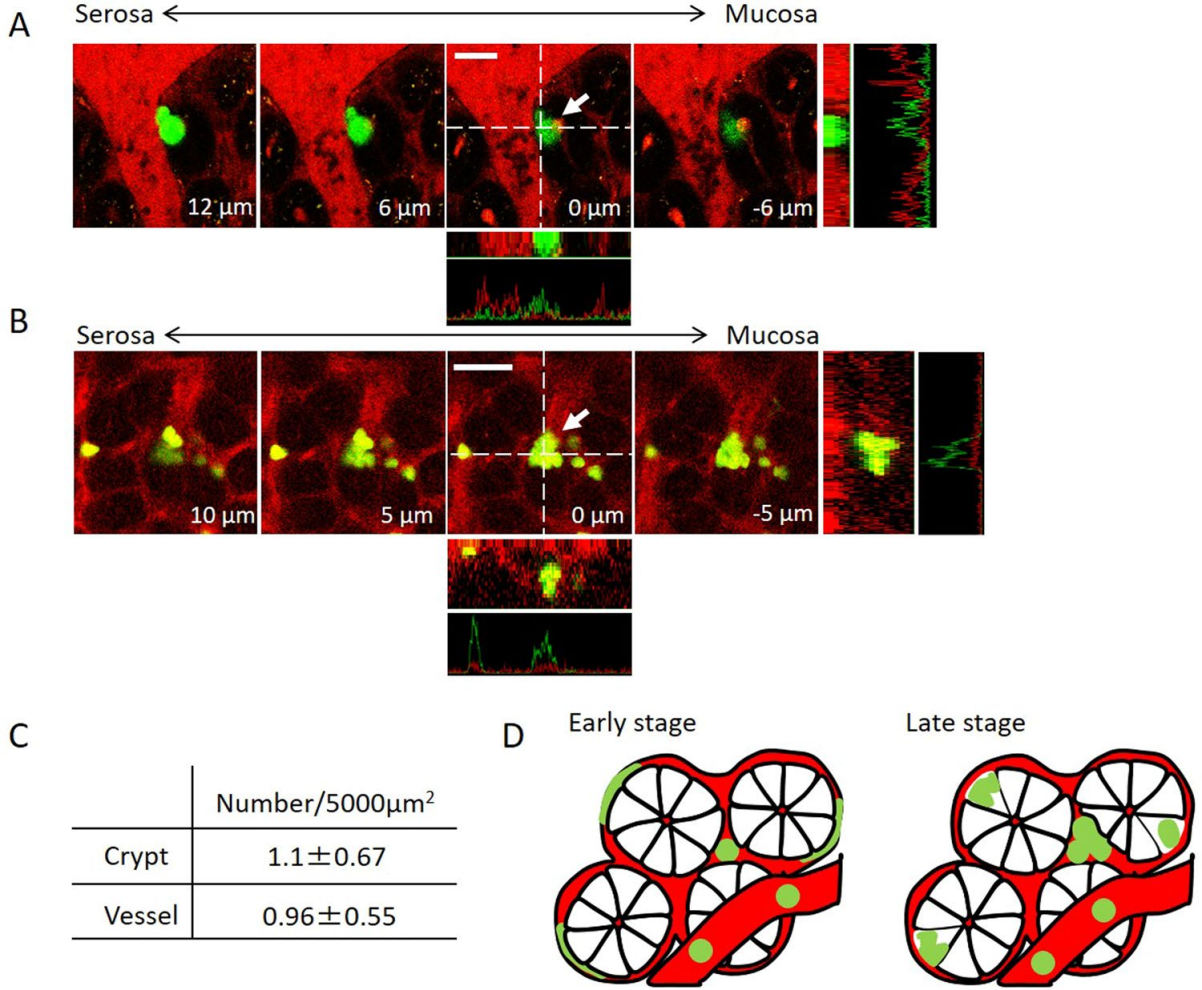
Formation of small nodules composed of several EL4-EGFP cells in crypts and blood vessels. (**A**) 3D image of a small nodule composed of EGFP-EL4 cells that infiltrated into crypts, as shown by a white arrow. Bar indicates 20 μm. (**B**) Image of a small EGFP-EL4 cell nodule in a blood vessel. Bar indicates 20 μm. This image is representative from 3 mice examined. (**C**). Percentage of EL4-EGFP nodules in crypts and blood vessels. (**D**). Schematic representation of the formation of nodules composed of EL4-EGFP cells. Green and red color indicate EL4-EGFP cells and blood vessels, respectively.

Finally, we investigated whether single EL4 cells are capable of forming assemblages. In the later stage after inoculation with EL4 cells (15–21 days), we observed sporadic extravasation of EL4 cells composed of more than single cells into the crypts from the adjacent blood vessels (Fig. 5A white arrow, Fig. 5C, and Supplementary Fig. 1A and B, white arrows). Notably, small nodules were also found in the blood vessels (Fig. 5B white arrow, Fig. 5C, and Supplementary Fig. 1C and D). These data indicate that EL4 cells lodged in the blood vessels in the mucosal layer can form small nodules that have the potential to form metastatic foci in the future (Fig. 5D), and that diverse mechanisms of nodule formation exist.

## Discussion

Our ultimate goal is to investigate where and how cancer cells infiltrate and form metastatic foci at the subcellular level *in vivo*. Toward this end, we established lines of EL4 cells that stably express EGFP or DsRed2 at high intensity and performed *in vivo* imaging using two-photon microscopy. Using this approach, after injection into mice, we observed EL4 cells lodged in blood vessels such as capillaries adjacent to colon crypts, which could not be detected with conventional HE staining. Whereas most EL4 cells were lodged in the small blood vessels, a proportion of the cells moved in an irregular manner along the blood vessels and interacted with other EL4 cells. This behavior could only be observed using the *in vivo* approach. In the later stages following injection of cancer cells, we discovered that the EL4 cells formed nodules in crypts and even in blood vessels, which could serve as the basis of establishing metastatic foci. Taken together, these results demonstrate that our system is suitable for *in vivo* analysis of the dynamics of cancer cell infiltration at subcellular resolution.

Upon the transport of cancer cells to specific organs via the blood circulation, small blood vessels such as capillaries (3–8 μm in diameter) serve as locations for cancer cells lodging^9^. A previous study in mice showed that after injection into the heart, HT-1080 cells enter the capillaries of the skin and become elongated^10^. Additionally, a series of studies reported that capillaries in the lung and liver play an important role in arresting circulating cancer cells^9,11,12^. In the present study, we found lodged and elongated EL4 cells in the small blood vessels in the colonic mucosal layer (Figs 1D and 2C). More than 70% of the EL4 cells were stabilized in the small blood vessels (Fig. 3C). Additionally, nodules of EL4 cells were observed in both the blood vessels and crypts in the later stages (Fig. 5A and B). There are two possibilities regarding how the small nodules of EL4 cells were formed. One is that a single EL4 cell divided to become an assemblage. The other possibility is that different cancer cells formed a clog. In the case of nodules of EL4 cells in blood vessels, they could be formed from different cells, as based upon our results showing variations in the fluorescence intensity of the cancer cells (Fig. 5B). By contrast, as the EL4 cells that had formed small nodules following extravasation from the blood vessels into the crypts exhibited the same fluorescence intensity, these cells likely derived from a single EL4 cell (Fig. 5A). These data indicate that there is diversity in the manner in which nodules are formed.

Most current research regarding cancer cell infiltration and metastasis is carried out using conventional HE staining of fixed tissue samples. However, this often fails to detect the infiltration of single cancer cells and micrometastasis. A number of imaging techniques have recently been employed in animal studies, including fluorescence imaging with visible and near-infrared wavelengths^13^, bioluminescence imaging^14^, and MRI and PET^15^. Another imaging technique, two-photon microscopy, enables in-depth analysis of the dynamics of cancer cell infiltration at subcellular resolution in living tissues including the brain, bone marrow, breast, and subcutaneous blood vessels^6,16–18^. In the present study, we utilized this advantage of two-photon microscopy to perform real-time imaging. We demonstrated the high motility potential of EL4 cells, even in small blood vessels (Fig. 3A and Supplementary Movies S1 and S2). Although our system did not reflect the reality of the first stage of cancer metastasis in terms of migration of cells to a primary site for infiltration and subsequent growth based on their escape and mobility, it did enable us to reveal with single-cell resolution details regarding the dynamics of cancer cells in the colon after they have become lodged at the site of infiltration. Therefore, this system could be used to observe the behavior of cancer cells in other organs, such as bone marrow, liver, and spleen.

Because the host immune system typically prevents the growth of most cancer cells, previous *in vivo* studies of cancer cell metastasis have relied on the use of immunodeficient mice, such as NOD/Shi-scid/IL-2Rγ^null^ and NOD.CB17-Prkdc^scid^/NcrCrl mice^19–22^. However, naturally occurring cancer cells proliferate and metastasize despite the host immune system. In this respect, it is more physiologically representative to use mice with an intact immune system for studying cancer cell dynamics. Although an excellent study examining mice with an intact immune system was recently published^23^, in the present study, we were able to inoculate EL4 cells that could be easily engrafted in C57BL6 mice. Using this model, we found that EL4 cells are capable of growing in both blood vessels and colon crypts (Fig. 5A and B). Thus, our system is more physiologically relevant for use in evaluating cancer cell growth in the presence of a functional immune system.

## Materials and Methods

Dulbecco’s Modified Eagle Medium (DMEM) was purchased from Nacalai tesque (Kyoto, Japan). 2-Mercaptoethanol, penicillin-streptomycin, and fetal bovine serum (FBS) were purchased from Life Technologies Japan, Ltd. (Tokyo, Japan). Rhodamine B isothiocyanate–dextran (M.W. 70,000) was from Sigma-Aldrich (St. Louis, MO, USA). Isoflurane was from Mylan Inc. (Canonsburg, PA, USA).

### Cells

EL4 mouse lymphoma cells were purchased from Japanese Collection of Research Bioresources. EL4, EL4-EGFP, and EL4-DsRed2 cells were maintained in DMEM supplemented with 10% heat-inactivated FBS, 55 μM 2-mercaptoethanol, 1% penicillin and streptomycin at 37 °C in a humidified 5% CO_2_ atmosphere.

### Establishment of cells stably expressing EGFP or DsRed2

EL4 cells were subjected to nucleofection using a 4D-Nucleofector™ System (Lonza, Japan) to transform EGFP or DsRed2 fluorescent protein. For EGFP nucleofection, the pmax-EGFP vector from Lonza (Sagamihara, Japan) was employed. For DsRed2 nucleofection, pCAG-DsRed2 prepared in our laboratory was used. One week after transfection of the plasmids encoding EGFP or DsRed2, EGFP- or DsRed2-positive cells were collected using a cell sorter (SH800, Sony). Three weeks after culture, EL4 cells stably expressing EGFP or DsRed2 were collected.

### Mice

C57BL/6 J and EGFP-expressing mice (C57BL/6-Tg[CAG-EGFP]) were purchased from CLEA Japan, Inc. (Tokyo, Japan). The mice were housed in the animal care facility under a 12-h light/12-h dark cycle at room temperature (23 ± 2 °C) and 55 ± 5% humidity. The mice were maintained on a diet of CE-2 rodent chow and given water *ad libitum*. The experimental protocols were reviewed and approved by the University of Tokyo Animal Ethics Committee. All efforts were done to minimize suffering and the number of mice used.

### Imaging in the colon

A total of 5 × 10^5^ EL4-EGFP and EL4-DsRed2 cells in 100 µl of PBS(−) were injected into C57BL6/J and C57BL/6-Tg(CAG-EGFP) mice, respectively, via the tail vein. A few weeks after injection, the mice were anesthetized with 2% isoflurane. The abdominal membrane was opened, the colon was removed from the body, and then the transverse and descending colon were observed under a two-photon microscope (FV1000 Olympus) with a 25x objective lens. Blood vessels were visualized with rhodamine B isothiocyanate–dextran (M.W. 70,000; 250 µg/ml, 100 µl) injected into the tail vein. Laser wavelengths were 880 nm for EGFP and 955 nm for DsRed2. 2–5 mW laser power under the objective lens was used to observe both EGFP and DsRed2. Images were acquired using GaAsP detectors.

### Histopathologic examination

Colon tissues were fixed in 4% paraformaldehyde and then subjected to standard tissue processing and paraffin embedding. The tissues were sliced serially into sections 3 μm thick for HE staining. Images were acquired microscopically (Keyence Corp., Japan). To detect immunofluorescence signals in unstained tissue sections, multispectral fluorescence analysis and spectral unmixing were performed using the Mantra quantitative pathology workstation and Inform software (PerkinElmer, Hopkinton, MA, USA).

### Ethics approval and consent to participate

This study does not contain human data and human tissue. For studies involving mice, the research protocol was accepted by the Ethical Committee for Animal Experiments of The University of Tokyo (approval ID: 28–5). We confirmed that all experiments were performed in accordance with relevant guidelines and regulations.

## Electronic supplementary material

Supplementary Information

Supplementary Movie S1 Real time imaging of EL4-EGFP cells in small blood vessels in adjacent to crypts in lamina propria of colon mucous membrane.

Supplementary Movie S2 Real time imaging of the interaction between EL4 cells in small blood vessels in colon.
