# Supplementary material for: In vivo imaging of T cell lymphoma infiltration process at the colon
Source: Sci Rep. 2018 Mar 5;8:3978. doi: 10.1038/s41598-018-22399-2 (PMC5838227; doi:10.1038/s41598-018-22399-2)
Supplement: Supplementary file 1 — Supplementary Information [file 41598_2018_22399_MOESM1_ESM.pdf]

Supplemental information

In vivo imaging of T cell lymphoma infiltration process at the colon

Yoshibumi Ueda[1,2,*], Toshiyuki Ishiwata[3], Seiichi Shinji[4], Tomio Arai[5], Yoko Matsuda[5], Junko Aida[3], Naotoshi Sugimoto[6], Toshiro Okazaki[7], Junichi Kikuta[8], Masaru Ishii[8], Moritoshi Sato[1]

[1] Graduate School of Arts and Sciences, The University of Tokyo, 3-8-1 Komaba, Meguro-ku, Tokyo 153-8902, Japan.

[2] AMED-PRIME, Japan Agency for Medical Research and Development, Tokyo, Japan.

[3]Division of Aging and Carcinogenesis, Research Team for Geriatric Pathology, Tokyo Metropolitan Institute of Gerontology, Tokyo 173-0015, Japan.

[4] Department of Gastrointestinal and Hepato-Biliary-Pancreatic Surgery, Nippon Medical School, Tokyo 113-8603, Japan.

[5] Department of Pathology, Tokyo Metropolitan Geriatric Hospital, Tokyo 173-0015, Japan.

[6] Department of Physiology, Graduate School of Medical Science, Kanazawa University, Kanazawa, Ishikawa, Japan.

[7] Department of Hematology and Immunology, Kanazawa Medical University, 1-1 Daigaku, Uchinada, Ishikawa 920-0293, Japan

[8] Department of Immunology and Cell Biology, Graduate School of Medicine and Frontier Biosciences, Osaka University, 2-2, Yamada-oka, Suita, Osaka, Japan

Supplementary figure 1

Other images for EL4 cell infiltration into to crypts and nodule formation in vessels at late stages

A and B. Other images of EL4-EGFP cells that were extravasated in crypts (white arrows). Red color exhibits blood vessels. White bars indicate 20 μm. C and D. Nodules of EL4-EGFP cells in blood vessels as shown in white arrows. Red color exhibits blood vessels. White bars indicate 20 μm.

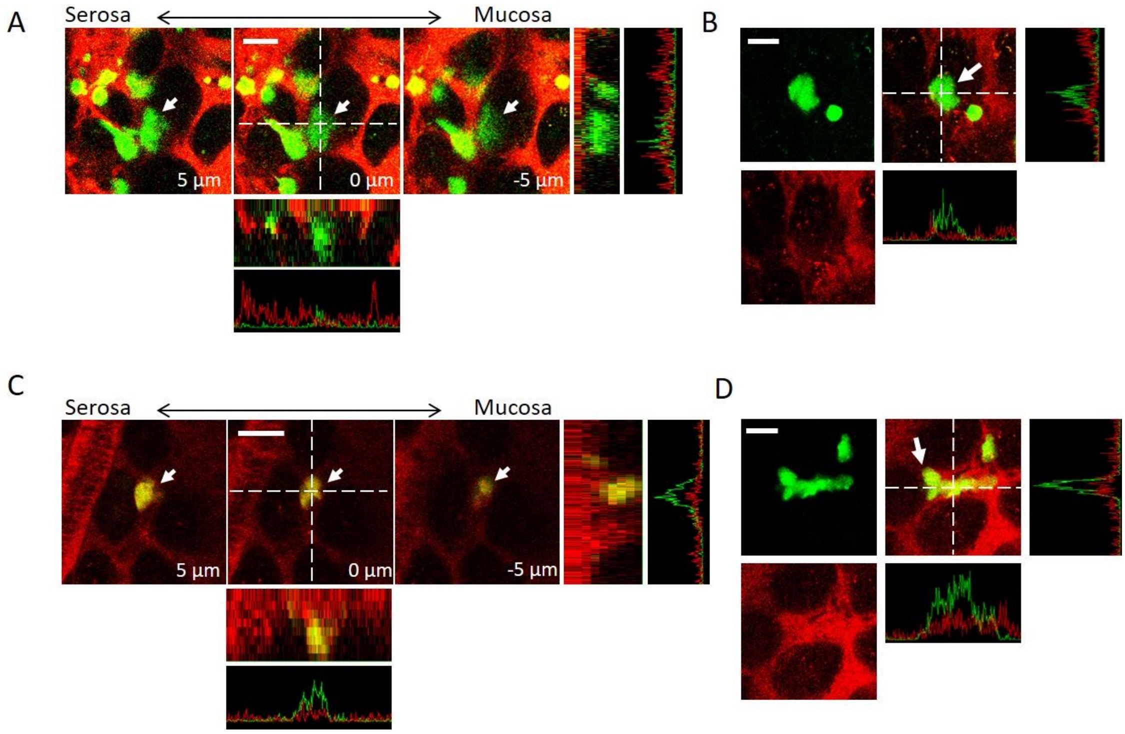

Supplementary data 2

Whole sequence of pCAG-DsRed2 vector.

DsRed2 sequence is highlighted with red color. HindIII, start codon, stop codon, and XhoI are colored with magenta, green, gray, and sky blue, respectively.

gacggatcgggagatctcccgatcccctatggtcgactctcagtacaatctgctctgatgccgcatagttaagccagtatctgct
ccctgcttgtgtgttggaggtcgctgagtagtgcgcgagcaaaatttaagctacaacaaggcaaggcttgaccgacaattgc
atgaagaatctgcttagggttaggcgttttgcgctgcttcggggctgcaggatttctagttattaatagtaatcaattacgggg
tcattagttcatagcccatatatggagttccgcgttacataacttacggtaaatggcccgcctggctgaccgcccaacgaccccc
gcccattgacgtcaataatgacgtatgttcccatagtaacgccaatagggactttccattgacgtcaatgggtggactatttac
ggtaaactgcccacttggcagtacatcaagtgtatcatatgccaagtacgccccctattgacgtcaatgacggtaaatggccc
gcctggcattatgcccagtacatgaccttatgggactttcctacttggcagtacatctacgtattagtcatcgctattaccatgg
gtcgaggtgagccccacgttctgcttcactctccccatctcccccccctccccacccccaattttgtatttatttattttttaattatt
ttgtgcagcgatgggggcgggggggggggggggcgcgcgccaggcggggcggggcggggcgaggggcggggcggggcga
ggcggagaggtgcggcggcagccaatcagagcggcgcgctccgaaagtttccttttatggcgaggcggcggcggcggcggc
cctataaaaagcgaagcgcgcggcgggcgggagtcgctgcgttgccttcgccccgtgccccgctccgcgccgcctcgcgccgcc
cgccccggctctgactgaccgcgttactcccacaggtgagcgggcgggacggcccttctcctccgggctgtaattagcgcttggt
ttaatgacggctcgtttcttttctgtggctgcgtgaaagccttaaagggctccgggagggccctttgtgcgggggggagcggct
cggggggtgcgtgcgtgtgtgtgtgcgtggggagcgccgcgtgcggcccgcgctgcccggcggctgtgagcgctgcgggcgcg
gcgcggggctttgtgcgctccgcgtgtgcgcgaggggagcgcggccgggggcggtgccccgcggtgcggggggggctgcgagg
ggaacaaaggctgcgtgcggggtgtgtgcgtgggggggtgagcagggggtgtgggcgcggcggtcgggctgtaacccccccc
ctgcacccccctccccgagttgctgagcacggcccggcttcgggtgcggggctccgtgcggggcgtggcgcggggctcgccgtg
ccgggcggggggtggcggcaggtgggggtgccgggcggggcggggccgcctcgggccggggagggctcgggggaggggc
gcggcggcccccggagcgccggcggctgtcgaggcgcggcgagccgcagccattgccttttatggtaatcgtgcgagagggcg
cagggacttcctttgtcccaaatctggcggagccgaaatctgggaggcgccgccgcaccccctctagcgggcgcgggcgaagc
ggtgcggcgccggcaggaaggaaatgggcggggagggccttcgtgcgtcgccgcgccgccgtccccttctccatctccagcctc
ggggctgccgcaggggggacggctgccttcgggggggacggggcagggcggggttcggcttctggcgtgtgaccggcggctct
agagcctctgctaaccatgttcatgccttcttctttttcctacagctcctgggcaacgtgctggttgttgtgctgtctcatcattttg
gcaaagaattgatttcgatacgtacggttatcaagcttGCCACCATGGCCTCCTCCGAGAACGTCATC
ACCGAGTTCATGCGCTTCAAGGTGCGCATGGAGGGCACCGTGAACGGCCACGAGT
TCGAGATCGAGGGCGAGGGCGAGGGCCGCCCCTACGAGGGCCACAACACCGTGA
AGCTGAAGGTGACCAAGGGCGGCCCCCTGCCCTTCGCCTGGGACATCCTGTCCCC
CCAGTTCCAGTACGGCTCCAAGGTGTACGTGAAGCACCCCGCCGACATCCCCGAC
TACAAGAAGCTGTCCTTCCCCGAGGGCTTCAAGTGGGAGCGCGTGATGAACTTCG

AGGACGGCGGCGTGGCGACCGTGACCCAGGACTCCTCCCTGCAGGACGGCTGCTT
CATCTACAAGGTGAAGTTCATCGGCGTGAACTTCCCCTCCGACGGCCCCGTGATGC
AGAAGAAGACCATGGGCTGGGAGGCCTCCACCGAGCGCCTGTACCCCCGCGACGG
CGTGCTGAAGGGCGAGACCCACAAGGCCCTGAAGCTGAAGGACGGCGGCCACTAC
CTGGTGGAGTTCAAGTCCATCTACATGGCCAAGAAGCCCGTGCAGCTGCCCGGCT
ACTACTACGTGGACGCCAAGCTGGACATCACCTCCCACAACGAGGACTACACCATC
GTGGAGCAGTACGAGCGCACCGAGGGCCGCCACCACCTGTTCCTGTAGctcgagcatgc
atctagagggccctattctatagtgtcacctaaatgctagagctcgctgatcagcctcgactgtgccttctagttgccagccatc
tgttgtttgcccctcccccgtgccttccttgaccctggaaggtgccactcccactgtcctttcctaataaaatgaggaaattgcatc
gcattgtctgagtaggtgtcattctattctggggggtggggtggggcaggacagcaagggggaggattgggaagacaatag
caggcatgctggggatgcggtgggctctatggcttctgaggcggaaagaaccagctggggctctagggggtatccccacgcg
ccctgtagcggcgcattaagcgcggcgggtgtggtggttacgcgcagcgtgaccgctacacttgccagcgccctagcgcccgct
cctttcgctttcttcccttcctttctcgccacgttcgccggctttccccgtcaagctctaaatcgggggcatccctttagggttccgatt
tagtgctttacggcacctcgaccccaaaaaacttgattagggtgatggttcacgtagtgggccatcgccctgatagacggtttt
tcgccctttgacgttggagtccacgttctttaatagtggactcttgttccaaactggaacaacactcaaccctatctcggtctatt
cttttgatttataagggattttggggatttcggcctattggttaaaaaatgagctgatttaacaaaaatttaacgcgaattaat
tctgtggaatgtgtgtcagttagggtgtggaaagtccccaggctccccaggcaggcagaagtatgcaaagcatgcatctcaat
tagtcagcaaccaggtgtggaaagtccccaggctccccagcaggcagaagtatgcaaagcatgcatctcaattagtcagcaa
ccatagtcccgcccctaactccgcccatcccgcccctaactccgcccagttccgcccattctccgccccatggctgactaattttttt
tatttatgcagaggccgaggccgcctctgcctctgagctattccagaagtagtgaggaggcttttttggaggcctaggcttttgc
aaaaagctcccgggagcttgtatatccattttcggatctgatcaagagacaggatgaggatcgtttcgcatgattgaacaag
atggattgcacgcaggttctccggccgcttgggtggagaggctattcggctatgactgggcacaacagacaatcggctgctct
gatgccgccgtgttccggctgtcagcgcaggggcgcccggttctttttgtcaagaccgacctgtccggtgccctgaatgaactgc
aggacgaggcagcgcggctatcgtggctggccacgacgggcgttccttgcgcagctgtgctcgacgttgtcactgaagcggga
agggactggctgctattgggcgaagtgccggggcaggatctcctgtcatctcaccttgctcctgccgagaaagtatccatcatg
gctgatgcaatgcggcggctgcatacgcttgatccggctacctgcccattcgaccaccaagcgaaacatcgcatcgagcgagc
acgtactcggatggaagccggtcttgtcgatcaggatgatctggacgaagagcatcaggggctcgcgccagccgaactgttc
gccaggctcaaggcgcgcatgcccgacggcgaggatctcgtcgtgacccatggcgatgcctgcttgccgaatatcatggtgga
aaatggccgcttttctggattcatcgactgtggccggctgggtgtggcggaccgctatcaggacatagcgttggctacccgtga
tattgctgaagagcttggcggcgaatgggctgaccgcttcctcgtgctttacggtatcgccgctcccgattcgcagcgcatcgcc
ttctatcgccttcttgacgagttcttctgagcgggactctggggttcgaaatgaccgaccaagcgacgcccaacctgccatcac
gagatttcgattccaccgccgccttctatgaaaggttgggcttcggaatcgttttccgggacgccggctggatgatcctccagcg
cggggatctcatgctggagttcttcgcccaccccaacttgtttattgcagcttataatggttacaaataaagcaatagcatcac
aaatttcacaaataaagcatttttttcactgcattctagttgtggtttgtccaaactcatcaatgtatcttatcatgtctgtatacc
gtcgacctctagctagagcttggcgtaatcatggtcatagctgtttcctgtgtgaaattgttatccgctcacaattccacacaac
atacgagccggaagcataaagtgtaaagcctggggtgcctaatgagtgagctaactcacattaattgcgttgcgctcactgcc

cgctttccagtcgggaaacctgtcgtgccagctgcattaatgaatcggccaacgcgcggggagaggcggtttgcgtattgggc
gctcttccgcttcctcgctcactgactcgctgcgctcggtcgttcggctgcggcgagcggtatcagctcactcaaaggcggtaat
acggttatccacagaatcaggggataacgcaggaaagaacatgtgagcaaaaggccagcaaaaggccaggaaccgtaaa
aaggccgcgttgctggcgtttttccataggctccgcccccctgacgagcatcacaaaaatcgacgctcaagtcagaggtggcg
aaacccgacaggactataaagataccaggcgtttccccctggaagctccctcgtgcgctctcctgttccgaccctgccgcttacc
ggatacctgtccgcctttctcccttcgggaagcgtggcgctttctcaatgctcacgctgtaggtatctcagttcggtgtaggtcgt
tcgctccaagctgggctgtgtgcacgaaccccccgttcagcccgaccgctgcgccttatccggtaactatcgtcttgagtccaac
ccggtaagacacgacttatcgccactggcagcagccactggtaacaggattagcagagcgaggtatgtaggcggtgctaca
gagttcttgaagtggtggcctaactacggctacactagaaggacagtatttggtatctgcgctctgctgaagccagttaccttc
ggaaaaagagttggtagctcttgatccggcaaacaaaccaccgctggtagcggtggtttttttgtttgcaagcagcagattac
gcgcagaaaaaaaggatctcaagaagatcctttgatcttttctacggggtctgacgctcagtggaacgaaaactcacgttaa
gggattttggtcatgagattatcaaaaaggatcttcacctagatccttttaaattaaaaatgaagttttaaatcaatctaaag
tatatatgagtaaacttggtctgacagttaccaatgcttaatcagtgaggcacctatctcagcgatctgtctatttcgttcatcc
atagttgcctgactccccgtcgtgtagataactacgatacgggagggcttaccatctggccccagtgctgcaatgataccgcga
gacccacgctcaccggctccagatttatcagcaataaaccagccagccggaagggccgagcgcagaagtggtcctgcaactt
tatccgcctccatccagtctattaattgttgccgggaagctagagtaagtagttcgccagttaatagtttgcgcaacgttgttgc
cattgctacaggcatcgtggtgtcacgctcgtcgtttggtatggcttcattcagctccggttcccaacgatcaaggcgagttaca
tgatcccccatgttgtgcaaaaaagcggttagctccttcggtcctccgatcgttgtcagaagtaagttggccgcagtgttatcac
tcatggttatggcagcactgcataattctcttactgtcatgccatccgtaagatgcttttctgtgactggtgagtactcaaccaa
gtcattctgagaatagtgtatgcggcgaccgagttgctcttgcccggcgtcaatacgggataataccgcgccacatagcagaa
ctttaaaagtgctcatcattggaaaacgttcttcggggcgaaaactctcaaggatcttaccgctgttgagatccagttcgatgt
aacccactcgtgcacccaactgatcttcagcatcttttactttcaccagcgtttctgggtgagcaaaaacaggaaggcaaaatg
ccgcaaaaaagggaataagggcgacacggaaatgttgaatactcatactcttcctttttcaatattattgaagcatttatcag
ggttattgtctcatgagcggatacatatttgaatgtatttagaaaaataaacaaataggggttccgcgcacatttccccgaaa
agtgccacctgacgtc

Supplementary Movie S1

Real time imaging of EL4-EGFP cells in small blood vessels in adjacent to crypts in lamina propria of colon mucous membrane.

EL4-EGFP cells were observed 10 days after the injection of $5x10^5$ EL4-EGFP cells into the tail vein in c57/BL6 mice. EL4-EGFP cells were intravitally imaged for 1 hr.

Supplementary Movie S2

Real time imaging of the interaction between EL4 cells in small blood vessels in colon.

EL4-EGFP cells were observed 10 days after the injection of $5x10^5$ EL4-EGFP cells into the tail vein in c57/BL6 mice. EL4-EGFP cells were intravitally imaged for 1 hr.
